# The role of social cognition skills and social determinants of health in predicting symptoms of mental illness

**DOI:** 10.1038/s41398-020-0852-4

**Published:** 2020-05-26

**Authors:** Hernando Santamaría-García, Sandra Baez, Carlos Gómez, Odir Rodríguez-Villagra, David Huepe, Maria Portela, Pablo Reyes, Joel Klahr, Diana Matallana, Agustin Ibanez

**Affiliations:** 1 Pontificia Universidad Javeriana, Physiology and Psychiatry Departments, Bogotá, Colombia; 2 Centro de Memoria y Cognición Intellectus, Hospital Universitario San Ignacio, Bogotá, Colombia; 3 Universidad de los Andes, Bogotá, Colombia; 4 Hospital Universitario San Ignacio, Epidemiology and Research Unit, Bogotá, Colombia; 5 Pontificia Universidad Javeriana, Epidemiology, Biostatistics and Psychiatry Departments, Bogotá, Colombia; 6 Institute for Psychological Research, University of Costa Rica, San Pedro, Costa Rica; 7 Neuroscience Research Center, University of Costa Rica, San Pedro, Costa Rica; 8 Center for Social and Cognitive Neuroscience (CSCN), School of Psychology, Universidad Adolfo Ibáñez, Santiago de Chile, Chile; 9 Family Medicine Section, Department of Emergency Medicine, George Washington School of Medicine and Health Sciences, Washington, DC USA; 10 Centro de Memoria y Cognición Intellectus, Hospital Universitario San Ignacio, Bogotá, Colombia; 11 Instituto de Envejecimiento, Hospital Universitario San Ignacio, Bogotá, Colombia; 12 Universidad de San Andrés, Buenos Aires, Argentina; 13 National Scientific and Technical Research Council (CONICET), Buenos Aires, Argentina; 14 Universidad Autónoma del Caribe, Barranquilla, Colombia; 15 Global Brain Health Institute (GBHI), University of California San Francisco (UCSF), San Francisco, CA USA

**Keywords:** Psychiatric disorders, Scientific community, Pathogenesis

## Abstract

Social factors, such as social cognition skills (SCS) and social determinants of health (SDH), may be vital for mental health, even when compared with classical psycho-physical predictors (demographic, physical, psychiatric, and cognitive factors). Although major risk factors for psychiatric disorders have been previously assessed, the relative weight of SCS and SDH in relation to classical psycho-physical predictors in predicting symptoms of mental disorders remains largely unknown. In this study, we implemented multiple structural equation models (SEM) from a randomized sample assessed in the Colombian National Mental Health Survey of 2015 (CNMHS, *n* = 2947, females: 1348) to evaluate the role of SCS, SDH, and psycho-physical factors (totaling 17 variables) as predictors of mental illness symptoms (anxiety, depression, and other psychiatric symptoms). Specifically, we assessed the structural equation modeling of (a) SCS (emotion recognition and empathy skills); (b) SDH (including the experience of social adversities and social protective factors); (c) and classical psycho-physical factors, including psychiatric antecedents, physical–somatic factors (chronic diseases), and cognitive factors (executive functioning). Results revealed that the emotion recognition skills, social adverse factors, antecedents of psychiatric disorders and chronic diseases, and cognitive functioning were the best predictors of symptoms of mental illness. Moreover, SCS, particularly emotion recognition skills, and SDH (experiences of social adversities, familial, and social support networks) reached higher predictive values of symptoms than classical psycho-physical factors. Our study provides unprecedented evidence on the impact of social factors in predicting symptoms of mental illness and highlights the relevance of these factors to track early states of disease.

## Introduction

Social processes are vital to achieving mental well-being^1,2^. Social and familial support, as well as social group membership, can positively impact resiliency and mental health^1^. In contrast, social discrimination or isolation can lead to stress, emotional suffering, and mental health problems^3^. In a broad sense, the social processes, which impact mental health encompass interactions between cognitive–personal factors and relational–contextual factors^3,4^. On the one hand, social cognition skills (SCS) are crucial to perceive and process relevant social cues in interactions with conspecifics^5–7^. SCS encompass the mechanisms involved in encoding, integrating, and behaving in the presence of socially relevant stimuli^6^. Critical among SCS are basic automatic processes (involving emotion and social cue perception) and more reflexive explicit processes (implicated in empathizing/mentalizing with others)^6,8^. SCS are considered as personal–cognitive factors affecting mental health. SCS have proven sensitive as transdiagnostic biomarkers of mental disorders^9–11^.

On the other hand, social determinants of health (SDH) include the conditions in which people are born, grow, live, work, and age according to the distribution of power and resources. SDH are considered contextual–relational factors potentially predicting health outcomes. Among SDH, both negative (violence experiences^7,12,13^, discrimination^14^, and social isolation^14^) and positive factors (social connectedness^15^, familial support^14^, and sense of belonging^15^) have proven crucial for mental health^16–19^.

Crucially, SCS and SDH seem to be intertwined^20^, as SDH vulnerability is associated with poor SCS^21^ and, reciprocally, reduced SCS increase SDH vulnerability^22^. For instance, different SDH, including the experiences of violence, discrimination, or isolation, are associated with enduring impacts on social and emotional cognitive processes^23,24^. Those experiences generate changes in stress responsivity impacting on neurocognitive processes, including emotional perception and regulation as well as empathic concern modulation^25–27^. In the opposite direction, impairments in specific SCS, such as individual predispositions for empathy and emotional recognition, can confer psychosocial risk, leading isolation, and dysfunctional coping styles that favor mental symptoms^28^. Aversive empathic reactions can encompass deviated cognitive perspective-taking and self-focused rumination, which facilitate the emergence of mental problems^29,30^.

Identifying and predicting symptoms of mental illness is one of the largest challenges of global health initiatives^31,32^. Anxiety, depression, and psychotic disorders are some of the most prevalent disorders with a major clinical impact on the general population^33^. These conditions lead to higher risks of suffering chronic mental illness, physical disorders^34,35^, increased number of comorbidities, poor chronic disease management^34,35^, decreased quality of life, and increased disability and mortality, and they have negative socioeconomic consequences^36^. In addition to the role of social factors in predicting mental health, other classical predictors have been described. Among the most classical predictors of symptoms of mental illness are various psycho-physical factors such as, previous psychiatric antecedents^35^, physical–somatic conditions^34,35^, and poor cognitive executive functioning^37^. Furthermore, convergent evidence highlights sex differences in the emergence of mental symptoms^38^. Risks for depressive and anxiety symptoms are higher in women than men^38^. Those effects in women have been associated with neurobiological correlates of social stress, differences in perception and regulation of emotional states, and social stress triggered by gender roles^38^. Aging is another crucial SDH that can impact SCS and the emergence of mental symptoms. Crucially, age is related to social challenges that modulate psychiatric symptoms peak in middle-aged and older individuals^34,35,38^.

Although both SCS and SDH are considered potential predictors of mental health, those factors have only been studied in isolation. In fact, a further understanding of the relative weight of those factors compared with other classical factors is still required. Against this background, we analyzed a comprehensive dataset from the adult population in the Colombian National Mental Health survey of 2015 (CNMHS, *N* = 2947). This dataset involves an unbiased and randomized approach and includes classical psycho-physical predictors, SCS, and SDH, as well as measures of symptoms of mental illness such as anxiety, depression and other psychiatric symptoms (illusions, delusions, hallucinations, and motor symptoms). We implemented a structural equation modeling (SEM) approach to test the predictive causal models of different outcomes and to estimate the causal relationships between groups of variables^39^, focusing on the prediction of symptoms of mental illness from SCS, SDH, and classical psycho-physical factors. We assessed five different SEMs: (a) an SCS-SEM (including emotion recognition and empathy skills); (b) an SDH-SEM (including social protective and social adverse factors); (c) a global social-SEM (combining SCS and SDH factors); (d) a classical psycho-physical-SEM (including psychiatric factors, physical–somatic factors and executive functioning scores); and (e) a global-integrated-SEM (integrating SCS, SDH, and classical psycho-physical factors).

Based on the hypothesis that social life has a strong impact on mind and health^6,9^, we anticipated significant prediction values and good model fit of SCS-SEM, SDH-SEM, and global social-SEM in predicting symptoms of mental illness. In addition, we expected the most accurate prediction of symptoms of mental illness by combining SCS, SDH, and classical psycho-physical factors rather than testing only the last. Furthermore, we anticipated reciprocal relationships between social and classical psycho-physical factors in predicting symptoms of mental illness.

## Materials and methods

### Data source and study sample

We analyzed data from the CNMHS, including individuals aged 18–59 years (mean age 42.6 years SD ± 16.5 years)^40^. The survey had a multistage cluster and probabilistic sample design, which provides representative data at the national and regional levels^41^. From every eligible sampled household, an individual aged ≥ 18 years was randomly selected and invited to participate. The survey collected data from a sample of 15,351 individuals, of which 10 870 were adults (18+ years). From the total sample, a subset of participants (*N* = 2947) was invited to participate in an interview, following a simple randomization method. This interview assessed: (a) SDH factors and (b) the presence of psychiatric antecedents and chronic diseases. In addition, they were assessed using cognitive and social cognition tasks, including (i) an emotion recognition task, (ii) an affective empathy task, and (iii) a battery to assess executive functions. Prior to completing this group of tasks, they provided an informed consent accepting to participate in this part of the study. The study was approved by the institutional ethics committee of the Pontificia Universidad Javeriana, Bogota, Colombia. All data were collected by a group of hired data collectors who did not know the purposes of this research. The researchers received the information collected anonymously. Survey details are provided in Supplementary material S1.

### Instruments

#### Symptoms of mental illness (outcome variable)

The presence of symptoms of mental illness were assessed by using a self-reporting questionnaire (SRQ)^42^. The SRQ-20 is a self-report assessment instrument of mental health administered via a paper/pencil questionnaire. The SRQ-20 was developed to assess depression, anxiety, and other psychiatric symptoms (illusions, delusions, hallucinations, and motor symptoms such as, tremors, myoclonus, and compulsive behavior). It has been found to be reliable and valid in different samples. Furthermore, it has proven to be robust for the screening of mental disorders in low- and middle-income countries, showing high internal reliability (Cronbach’s *α* = 0.84)^42^. Item responses are recorded as binary (yes = 1, no = 0) and cover a 30-day recall period. Summing the individual items gives a maximum total score of 20. The number of positive symptoms for each of the three categories was used to create three item parcels for the latent Mental symptoms as outcome variables.

### SCS

#### Emotion recognition task

We assessed facial emotion recognition using the emotion recognition task (EMT)^43^, which comprises photos of facial expressions featuring six basic emotions (happiness, surprise, sadness, fear, anger, and disgust). We used 12 facial stimuli depicting these basic emotions. We measured the mean accuracy of overall emotion recognition (maximum one point) and the accuracy of each emotion category. To facilitate the analyses of emotion recognition skills, we built two item parcels measures following previous studies^44^: one based on the average of emotion recognition of positive and neutral valence faces (happiness and surprise, neutral) and another based on the average of emotion recognition of negative valence faces (sad, disgust, and fear)^45^. This task has been previously validated^46^, is robust in tracking deficits in patients with neuropsychiatric disorders^9^ and has been used in other population-based studies^44^ (Supplementary material S2.1).

#### Empathy for pain task (EPT)

We used a modified version of a previously reported EPT^47^, which evaluates various dimensions of empathy in situations containing intentional or accidental harm. The EPT comprises 11 animated scenarios (4 intentional, 4 accidental, and 3 neutral) involving two individuals. This task evaluates different empathy domains, including (a) comprehension of the accidental or deliberate nature of the action and the intention of the perpetrator to hurt, (b) the empathic concern, (c) the degree of discomfort for the victim, (d) the detection of intentional harm ascribed to the perpetrator, and (e) the punishment deserved by the perpetrator. Following a previous protocol^48^, these measures were used to assess both affective (averaging the scores of empathic concern and discomfort) cognitive (averaging the scores of intention to harm and punishment) aspects of empathy. Both affective and cognitive empathy measures were created for intentional and accidental situations. This task has been used in neuropsychiatric populations and in population-based studies^48^. For a further description of the EPT see Supplementary material S2.2.

### SDH

#### Social adverse and social protective factors

Regarding adverse factors, the presence of experiences of discrimination, violence and social stress associated with reduced access to social resources was assessed by using yes/no questions. Regarding protective factors, social support networks were assessed by asking individuals about their participation in different social groups using yes/no questions (Supplementary material S3.1–S3.2). A sum of positive answers in the aforementioned factors was used to build parcels with these observable variables for SEM analyses. In addition, family support was assessed using the Family Apgar instrument^49^, which measures family functioning according to five factors: adaptation, companionship, development, affectivity, and problem-solving ability. The score was 0 when participants answered a question negatively and 2 when they reported affirmatively. High scores indicate major family support^49^ (Supplementary material S3.2).

### Classical psycho-physical factors

#### Psychiatric antecedents

Participants were assessed on whether they have been diagnosed during their lifespan with a general psychiatric disease but also, whether they have been diagnosed with an affective diseases. To this end, we used the Composite International Diagnostic Interview (CIDI), which follows the criteria of the Diagnostic and Statistical Manual of Mental Disorders (Fifth Edition, Text Revision: DSM-IV-R). The presence of general psychiatric and affective diseases were coded using yes/no answers (yes = 1, no = 0). Thus, we measured two observable variables (a) antecedents of general psychiatric disorders and (b) antecedents of affective disorders. A sum of positive answers in the mentioned variables was used to build parcel of both observable variables to test in the SEMs (Supplementary material S4).

#### Physical–somatic factors (chronic somatic diseases)

Participants indicated the presence or absence of relevant chronic diseases, including arterial hypertension, diabetes mellitus, obesity, heart-vascular disease, brain-vascular disease, arthritis, hypothyroidism symptoms, and infectious diseases (particularly antecedents of herpes zoster, syphilis, HIV-AIDS, and tuberculosis). This variable was assessed using yes/no questions and ranged from 0 (absence of any chronic disease) to 11 (presence of all aforementioned diseases). A sum of positive answers in this variable was used to build parcel of this observable variable to test in the SEMs.

#### Cognitive functioning

All participants were evaluated using an executive function battery^50^ measuring (1) motor programming, (2) conflicting instructions, (3) verbal inhibitory control, and (4) numerical working memory (backward digit span). These subtasks have been used to successfully detect executive function in clinical^51^ and nonclinical populations^51^. The total score of each subtask ranged from 0 to 3. The 12 score was assigned when participants answered the items of each subtask appropriately (Supplementary material S5). A mean of scores in the measures described was used to build parcels of this observable variable to test in the SEMs.

#### Assessment of covariables (demographic factors)

**Fig. 1:**
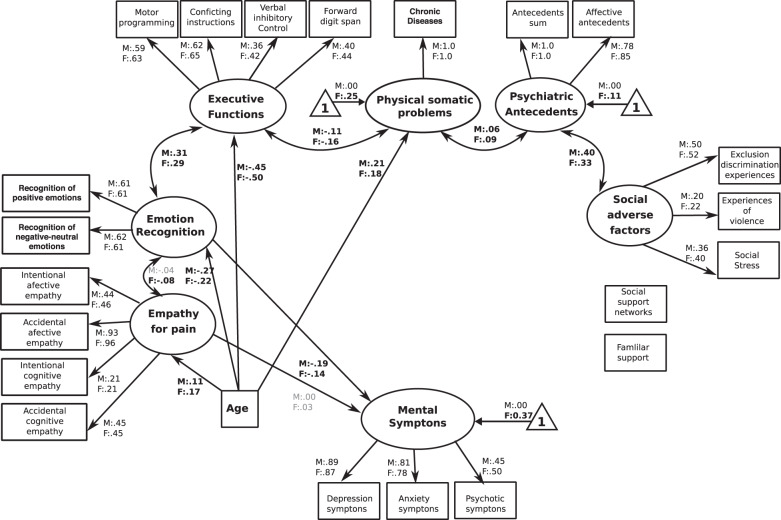
The SCS-SEM. The figure reveals the path regressors of SCS as predictors of symptoms of mental illness. Circles depict latent (unobserved theoretically built) variables. Squares depict observable (measured) variables. Directional arrows depict direct effect of one variable over another (regressor paths). Bidirectional arrows reveal no directional association between two latent variables. Bold numbers indicate significant (standardized) regressor scores. The outcome of latent and observable variables (symptoms of mental illness) is shown in gray. Triangles with a “1” on the interior and including an arrow pointing to a specific latent variable depicts the latent intercepts (i.e., factor means). The figure only shows latent intercepts in which the estimated factor mean of females was statistically different from males. For ease representation error terms are not shown in the figure.

**Fig. 2:**
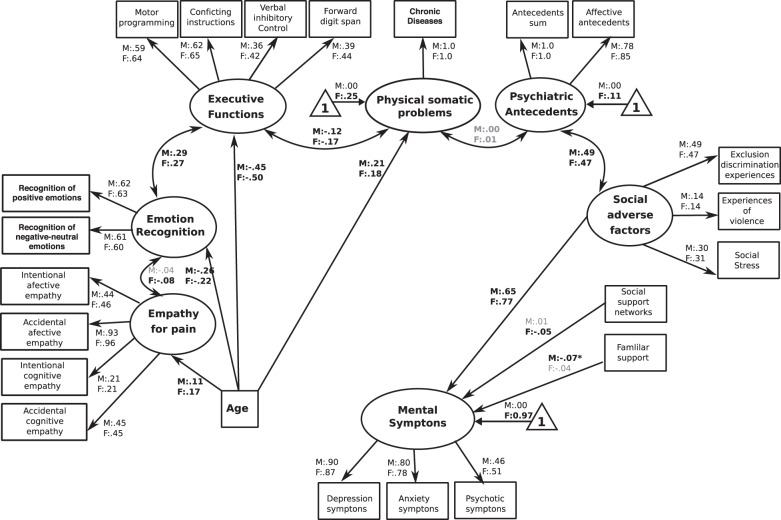
The SDH-SEM. The figure reveals the path regressors of SDH as predictors of symptoms of mental illness. Circles depict latent (unobserved theoretically built) variables. Squares depict observable (measured) variables. Directional arrows depict direct effect of one variable over another (regressor paths). Bidirectional arrows reveal no directional association between two latent variables. Bold numbers indicate significant (standardized) regressor scores. The outcome of latent and observable variables (symptoms of mental illness) is shown in gray. Triangles with a “1” on the interior and including an arrow pointing to a specific latent variable depicts the latent intercepts (i.e., factor means). The figure only shows latent intercepts in which the estimated factor mean of females was statistically different from males. For ease representation error terms are not shown in the figure.

**Fig. 3:**
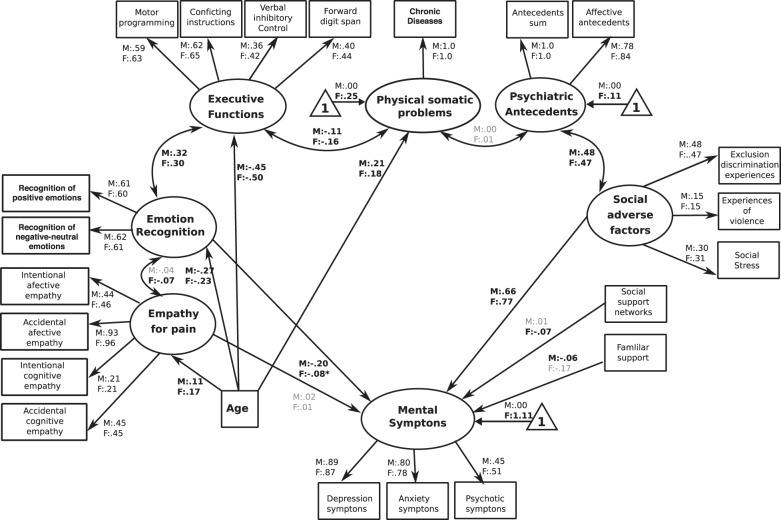
The global social-SEM. The figure reveals the path regressors of SCS and SDH factors in predicting symptoms of mental illness. Circles depict latent (unmeasured theoretically built) variables. Squares depict observable (measured) variables. One-way bold arrows depict regressor paths. Two-way arrows reveal significant covariation among latent variables. Bold numbers indicate significant (standardized) regressor scores. The outcome of latent and observable variables (symptoms of mental illness) is shown in gray. Triangles with a “1” on the interior and including an arrow pointing to a specific latent variable depicts the latent intercepts (i.e., factor means). The figure only shows latent intercepts in which the estimated factor mean of females was statistically different from males. For ease representation error terms are not shown in the figure.

**Fig. 4:**
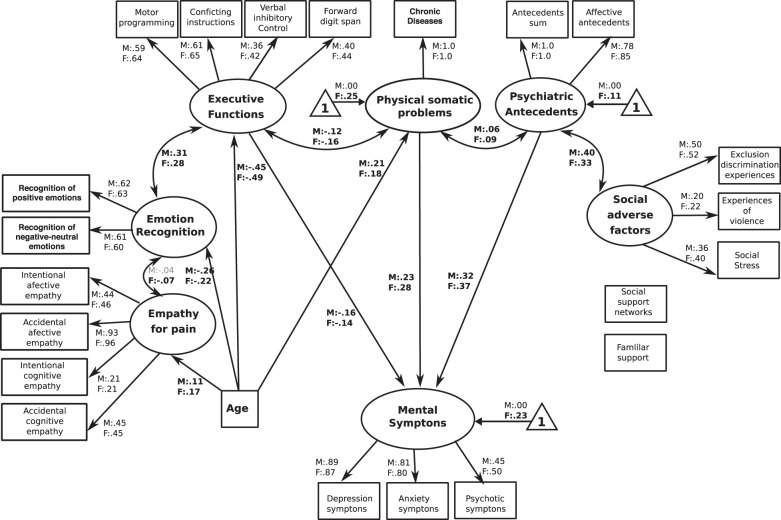
The classical psycho-physical-SEM. The figure reveals the path regressors of different psycho-physical factors, including the presence of psychiatric antecedents, somatic problems and cognitive functioning. Circles depict latent (unmeasured theoretically built) variables. Squares depict observable (measured) variables. One-way bold arrows depict regressor paths. Two-way arrows reveal significant covariation among latent variables. Bold numbers indicate significant (standardized) regressor scores. The outcome of latent and observable variables (symptoms of mental illness) is shown in gray. Triangles with a “1” on the interior and including an arrow pointing to a specific latent variable depicts the latent intercepts (i.e., factor means). The figure only shows latent intercepts in which the estimated factor mean of females was statistically different from males. For ease representation error terms are not shown in the figure.

**Fig. 5:**
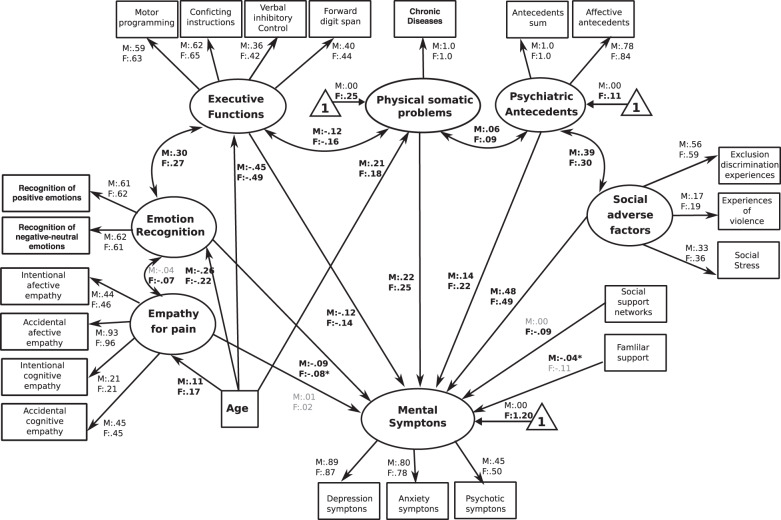
The global-integrated-SEM. This figure shows the path regressors of an integrated combination of possible predictors of symptoms of mental illness, including the SCS, the SDH factors, and the presence of psychiatric antecedents, the presence of psychical–somatic problems and cognitive functioning. The figure shows that emotion recognition skills (SCS), the presence of social adverse factors and social protective factors (both factors belonging to SDH), the presence of psychiatric antecedents and the presence of physical–somatic problems significantly predict symptoms of mental illness. Circles depict latent (unmeasured theoretical built) variables. Squares depict observable (measured) variables. One-way bold arrows depict regressor paths. Two-way arrows reveal significant covariation between latent variables. Bold numbers indicate significant (standardized) regressor scores. The outcome of latent and observable variables (symptoms of mental illness) is shown in gray. Triangles with a “1” on the interior and including an arrow pointing to a specific latent variable depicts the latent intercepts (i.e., factor means). The figure only shows latent intercepts in which the estimated factor mean of females was statistically different from males. For ease representation error terms are not shown in the figure.

All individuals fulfilled general demographic information assessing age (years) and sex (females (F) and males (M)). Demographic information was used as covariates in all SEM analyses (Tables 1, 2 and Figs. 1–5).

**Table 1:** Descriptive analysis of measures for different predictors of mental problems.

Variables	Statistical values Percentage of cases mean scores Females/males (F:M)
Participants	*n* = 2947
Sex (F:M)	*n* = 1348: *n* = 1599
Age [mean (SD)] (F:M)	43.02 (16.5):42.32 (16.5)
Mean educational level (SD in years) (F:M)	5.4 (1.5):5.4 (1.9)
Assessment of social cognition skills (SCS) (F:M)	
Total percentage of face emotion recognition	62% (17%):62.3% (17%)
Percentage of negative emotion recognition	46.7% (21%):45.2% (22%)
Percentage of positive emotion recognition	79.7% (22%):81.6% (22%)
Mean scores of affective empathy in intentional scenarios	5.5 (1.5):5.2 (1.5)
Mean scores of affective empathy in accidental scenarios	4.9 (1.3):5.0 (1.3)
Mean scores of cognitive empathy in intentional scenarios	6.6 (1.3):6.5 (1.3)
Mean scores of cognitive empathy in accidental scenarios	4.9 (1.5):4.8(1.4)
Assessment of social determinants of health (SDH) (F:M)	
Social adverse factors (mean of experiences of discrimination and isolation)	0.7 (1.3):0.6 (1.3)
Social adverse factors (mean of experiences of violence)	1.2 (0.6):1.2 (0.6)
Social adverse factors (mean of experiences of social isolation)	4.4 (1.6):4.6 (1.6)
Social protective factors (mean scores of family APGAR)	9.2 (8.9):9.4 (9.0)
Social protective factors (mean of number of the participation in social groups)	0.43 (0.63):0.45 (0.69)
Assessment of psycho-physical factors	
Psychiatric antecedents (presence of general psychiatric antecedents across life)	0.18 (0.68):0.11 (0.47)
Psychiatric antecedents (presence of affective psychiatric antecedents across life)	0.06 (0.33):0.03 (0.24)
Psychical–somatic problems (mean of number of somatic symptoms)	5.6 (1.1):5.4 (1.0)
Cognitive functioning (mean of motor programing task)	2.3 (0.7):2.3 (0.7)
Cognitive functioning (mean of conflicting instructions tasks)	2.5 (0.6):2.6 (0.6)
Cognitive functioning (mean of inhibitory verbal control task)	3.3 (2.1):3.4 (2.1)
Cognitive functioning (mean of scores of the backwards digit span task)	2.8 (1.0):3.0 (0.9)
Assessment of symptoms of mental illness	
Presence of depression symptoms	1.2 (1.8):0.7 (1.4)
Presence of anxiety symptoms	1.4 (1.7):0.7 (1.3)
Presence of other symptoms (convulsions, sensorial perceptual symptoms etc.)	0.9 (0.9):0.7 (0.8)

**Table 2:** Parameters of goodness-of-fit of the different structural equation models SEM.

SEM	YB *χ*^2^	Df	*P*	Robust CFI	Robust RMSEA	AIC	Sample-size-adjusted Bayesian (aBIC)
1. The global-integrated model-SEM	1235.2	410	<0.00	0.93	0.036	143,509	143,852
2. The SCS/SDH-SEM (global social model)	1376.2	416	<0.00	0.91	0.041	143,751	144,066
3. The classical psycho-physical factors-SEM	1385.4	420	<0.00	0.91	0.041	143,752	144,076
4. Social determinants of Health (SDH)-SEM	1442.7	420	<0.00	0.91	0.042	143,817	144,131
5. Social cognition skills (SCS)-SEM	1919.5	422	<0.00	0.86	0.049	144,344	144,653

The models are presented in order based on its goodness-of-fit.

### Analysis methods

#### SEM procedures

SEM is a hybrid technique that includes aspects of confirmatory factor analysis, path analysis and regression for building and testing the predictive causal models of different outcomes and estimates the causal relationships among groups of variables^39^ (Supplementary material S6). Observed variables (variables that are measured directly and are described at the Instrument and Supplementary material S7) were used and tested (using significant regressor scores as predictors) to build latent (unobserved) variables. As the data were not normally distributed, we used maximum likelihood estimation with robust (Huber–White) standard errors. The chi-square (*χ*^2^) statistic was scaled by Yuan–Bentler correction factor (YB). Full information maximum likelihood estimation method was implemented to handle missing data. Structural equation modeling adequacy fit indices were used to assess model goodness-of-fit: the YB *χ*^2^ statistic, the robust comparative Fit index (robust CFI), and the root mean square error approximation (robust RMSEA)^52^.

Following a previous procedure^53^, we derived different individual SEMs from a single SEM integrating all predictors of mental symptoms. For each individual model, we tested a selective set of factors in predicting mental symptoms and fixed to zero values all other factors. Thus, we tested five different SEMs: (a) an SCS-SEM (analyzing only the predictive scores of emotion recognition and empathy skills with the rest of parameters fixed to 0); (b) an SDH-SEM (analyzing only the predictive scores of social protective and social adverse factors with the rest of parameters fixed to 0); (c) a global social-SEM (analyzing the predictive scores of SCS and SDH factors and the rest of parameters fixed to 0); (d) a classical psycho-physical-SEM (analyzing the predictive scores of psychiatric factors, physical–somatic factors and executive functioning scores with the social factors fixed to 0); and (e) a global-integrated-SEM (analyzing together the SCS, SDH and classical psycho-physical factors). This procedure allowed us to compare the goodness-of-fit of models assuming the same number of factors in all models and the same number of relationships between them. Akaike and sample-size adjusted Bayesian (aBIC) criteria were used to compare the goodness-of-fit indices between models (Table 2). Data processing and all analyses were made using R.

## Results

Table 1 summarizes the detailed results, including means, percentages, and standard deviations of each SCS, SDH, and classical psycho-physical factor. In addition, Table 2 provides the parameters of goodness-of-fit of the various SEM models.

## The SCS-SEM

This model showed significant fit to data [YB *χ*^2^(410) = 1919.52, *P* < 0.001, robust CFI = 0.86, robust RMSEA = 0.049 (90% confidence interval [CI] = 0.048–0.050)]. The standardized regression coefficients (regression of one variable on another) revealed that symptoms of mental illness were negatively predicted by the emotion recognition scores in both sexes (F:M = −0.14:−0.19, *P* < 0.001). The latent variables emotion recognition and empathy for pain were correlated for females (*F* = 0.07, *P* < 0.05) but not for males (*F* = 0.04, *P* = 0.24). In addition, emotion recognition was positively correlated to executive functioning in both sexes (F:M = 0.29:0.31, *P* < 0.001). Furthermore, age was negatively correlated with emotion recognition (F:M = −0.22:−0.27, *P* < 0.001), and positively correlated with empathy for pain (F:M = 0.17:0.11, *P* < 0.001). No other factors significantly covariate with regressors (Fig. 1).

## The SDH-SEM

Analyses of this model showed significant fit to data [YB *χ*^2^(420) = 1442.71, *P* < 0.001, robust CFI = 0.91, robust RMSEA = 0.042 (90% CI = 0.040–0.045)]. All factor loadings were statistically significant at *P* < 0.001. The standardized regression coefficients showed that mental illness symptoms were positively predicted by the presence of adverse factors in both sexes (F:M = 0.65:0.77, *P* < 0.0001), as well as negatively predicted by the social support network in females (−0.05, *P* < 0.001) and by familial support in males (−0.07, *P* < 0.001). Furthermore, we observed a positive correlation between latent variable of social adverse factors and psychiatric antecedents in both sexes (F:M = 0.47:0.49, *P* < 0.001, Fig. 2).

## The global social-SEM

The analyses of this model showed significant indices of fit [YB *χ*^2^(416) = 1376.20, *P* < 0.001, robust CFI = 0.91, robust RMSEA = 0.040 (90% CI = 0.039–0.044)]. The standardized regression coefficients revealed that the symptoms of mental illness were positively predicted by social adverse factors (F:M = 0.77:0.66, *P* < 0.0001) and negatively predicted by emotion recognition (F:M = −0.08:−0.20, *P* < 0.001); by the presence of social support networks in females (−0.07, *P* < 0.001) and by familiar support in males (−0.06, *P* < 0.001). Moreover, the results revealed other associations, as emotion recognition was positively correlated with executive functioning (F:M = 0.30:0.32, *P* < 0.001) and with empathy for pain in females (0.07, *P* < 0.001). Moreover, the latent variable of social adverse factors was positively correlated to psychiatric antecedents (F:M = 0.47:0.48, *P* < 0.001, Fig. 3).

## The classical psycho-physical-SEM

The analyses of this model showed significant indices of fit [YB *χ*^2^(420) = 1385.42, *P* < 0.001, robust CFI = 0.916, robust RMSEA = 0.041 (90% CI = 0.039–0.043)]. In this case, regression coefficients showed that symptoms of mental illness were positively predicted by physical–somatic problems (F:M = 0.28:0.23, *P* < 0.001) and by psychiatric antecedents (F:M = 0.37:0.31, *P* < 0.001). Furthermore, they were negatively predicted by executive functioning (F:M = −0.14:−0.16, *P* < 0.001). Age was positively correlated with physical–somatic problems (F:M = 0.18:0.21, *P* < 0.001) and negatively correlated with executive functioning (F:M = −0.49:−0.44, *P* < 0.001). Furthermore, physical–somatic symptoms were positively associated to psychiatric antecedents (F:M = 0.09:0.06, *P* < 0.001) and negatively associated to executive functioning (F:M = −0.16:−0.12, *P* < 0.001, Fig. 4).

## The global-integrated-SEM

The model reached significant values and showed the highest fit indexes in comparison to the previous models [YB *χ*^2^(410) = 1144.10, *P* < 0.001, robust CFI = 0.93, robust RMSEA = 0.036 (90% CI = 0.034–0.039)]. Regression coefficients showed that symptoms of mental illness were positively predicted by the presence of social adverse factors (F:M = 0.49:0.48, *P* < 0.0001), physical–somatic problems (F:M = 0.25:0.22, *P* < 0.0001), and psychiatric antecedents (F:M = 0.22:0.14, *P* < 0.001). Furthermore, symptoms of mental illness were negatively predicted by emotion recognition of SCS (F:M = −0.09:−0.08, *P* < 0.01), by executive functioning (F:M = −0.14:−0.12, *P* < 0.001) and by social support networks in females (−0.09, *P* < 0.01) and family support in males (−0.04, *P* < 0.01). Age was negatively associated with executive functions (F:M = −0.49:−0.45, *P* < 0.0001), with emotion recognition (F:M = 0.25:0.23, *P* < 0.001), but positively correlated with physical–somatic problems (F:M = 0.18:0.21, *P* < 0.0001), and with empathy for pain (F:M = 0.17:0.11, *P* < 0.01). As in the previous models, a similar pattern of correlations between latent psycho-physical, SCS and SDH variables was observed. Physical–somatic problems were positively associated to psychiatric antecedents and negatively associated to executive functioning. Executive functioning was positively correlated to emotion recognition; and psychiatric antecedents were positively associated to social adverse factors (Fig. 5 and Supplementary Table 1).

## SEM comparison

The conventional fit indices and the AIC and aBIC criteria revealed that the best-fit model among the five SEMs was the global-integrated model. Moreover, the second model with highest fit indexes was the global social-SEM, which reached better fit scores than the classical psycho-physical-SEM. Finally, we found the SDH and SCS SEMs, in particular, the SDH-SEM reached higher fit indexes than SCS-SEM (Table 2).

## Discussion

In this randomized probabilistic design, we evaluated to what extent a combined set of social factors (SCS and SDH) is able to predict symptoms of mental illness. To our knowledge, this is the first study to highlight the importance of a broad range of social factors (internal factors such as SCS and contextual factors such as SDH) in combination with classical factors in predicting the presence of a broad spectrum of symptoms of mental illness, which can be considered as subclinical states of mental disorders. Our results revealed that SEMs using SCS and SDH reached high fit values and showed that those factors are able to predict symptoms of mental illness. Moreover, our results showed that the integrated social model (SCS and SDH) reached even a higher prediction scores of symptoms of mental illness compared with the model using classical predictors.

The SEM analysis of SCS revealed that individuals with better scores on the emotion recognition task exhibited lower scores of symptoms of mental illness. This effect was modulated by age. Furthermore, in agreement with previous studies^43,47^, this model showed an association between emotion recognition and executive functioning in both sexes. Our results are in line with studies showing poor emotion recognition in individuals with poor executive functioning^51^, patients with depressive and anxiety disorders^9^, and individuals with other symptoms, including psychotic symptoms^9,54^. Individuals with symptoms of mental illness tend to bias their attentional resources focus those resources on threatening stimuli and to perceive or interpret neutral faces as negative emotions (anger, fear, or disgust)^9^. The SCS-SEM found no significant association among empathy and symptoms of mental illness. Although previous studies shown differences in empathy for pain skills in case–control designs in different psychiatric and neurological populations^9,55–67^, no studies have revealed that individual differences in empathy for pain can predict mental symptoms. However, as in previous reports^9,68^, empathy correlated with emotion recognition in females. Arguably, the preponderant predictive effect of emotion recognition in mental symptoms manifestation could mask the role of empathy in predicting mental symptoms. In fact, emotion recognition seems to be a more consistent biomarker of mental illness than empathy^9^. Future studies should analyze to what extent emotion recognition interact with empathy to predict mental symptoms.

The SDH-SEM showed high fit indexes and evidenced that higher scores of social adverse factors (past experiences of discrimination, poor social access, and violence) and lower scores of family support in males and reduced participation in social groups in females predicted a major presence of symptoms of mental illness. Our results coincide with cumulative evidence^16,17,31^ revealing that developmental and life exposure to violence, discrimination and social exclusion predicts symptoms of mental illness and disruptive behaviors. Arguably, social adversities can increase the risk of suffering from a mental illness due to generation of insecure or unconfident attachment styles, modification of genetic-epigenetic interactions^69^, and changes in affective self-regulation^69^. Our results support these evidences as they revealed positive associations between social adverse factors and psychiatric antecedents. By contrast, experience of familial support and a sense of belonging generate flexible and enriched relational patterns^21^, promote affective regulation and modulate empathic skills^22^. Here, we show that the assessment of SDH yields a consistent pattern of prediction of symptoms of mental illness and those effects are modulated by sex.

The model integrating SCS and SDH reached higher fit indexes than classical psycho-physical-SEM (Table 2) and revealed that increased scores of social adverse factors and reduced scores of emotion recognition skills were the most predictive factors of symptoms of mental illness. Our results also showed that an integrative assessment of both SCS and SDH reached a better fit index than the independent SCS-SEM and SDH-SEM. Although previous studies have shown that SCS and SDH seems to be intertwined^21,22^, no studies had assessed their integrated role in predicting symptoms of mental illness. Our study adds new information to past evidence, revealing the necessity of assessing SCS and SDH to increase predictions of mental symptoms.

The classical psycho-physical-SEM evidenced that symptoms of mental illness were associated with antecedents of psychiatric disorders, poor executive functioning, and the presence of chronic diseases. Again, those effects were modulated by age. Previous studies have reported that separately these factors (psychiatric antecedents, chronic diseases, and cognitive skills) are associated with symptoms of mental illness^34,35,37^. The results of this study add new evidence to this field by revealing to what extent these classical factors predict mental symptoms when they are assessed in combination, as occurs in more complex models of mental health.

The sources of mental problems probably unveil complex interactions among demographic, psychiatric, somatic, and social factors^9^. The results of different SEMs in this study revealed that the complex combination of factors better captured the presence of mental symptoms than models using isolated groups of predictors. The globally integrated SEM revealed the best goodness-of-fit scores compared to other SEMs (Table 2). This integrated model revealed that the most relevant predictors of symptoms of mental illness also have a relevance ranking, from the presence of social adversities and to a lesser extent the chronic diseases, psychiatric antecedents, executive dysfunction, and low emotion recognition scores. Furthermore, the mental symptoms were also predicted by the quality of family and social support networks and those effect were mediated by sex. To our knowledge, this is the first study to assess in an integrated way different types of predictors of symptoms of mental illness. Crucially, the model suggests that the emergence of mental symptoms is a multidependent phenomenon associated with different levels of vulnerability that rely on the complex interactions of social, psychological, and physical factors.

Our study has some limitations that call for further assessments. Using self-reported measures in mental illness research is a common limitation^2,70^ that could underestimate or overestimate different predictors due to recall bias. However, we combined self-reported measures with other standardized and experimental measures and both types reached similar regression scores in the SEM. Future assessment of SDH associated with symptoms of mental illness should confirm our results by using less obtrusive measurement strategies such as scales tracking social adverse factors and other randomized response techniques. A robust randomization process was utilized to decrease the probability of selection bias; however, our subset battery of analyses was conducted on the adult population only and thus is limited in its generalizability to the overall population. An extra consideration is that our results were obtained from a low- and middle-income country (Colombia). Future research should test the stability and generalizability of the present results.

New studies should further evaluate the relationship between SCS, mental symptoms, and other groups of SDH, including literacy, educational opportunities, intelligence, food and nutritional antecedents, and economic resources stability. Some studies have revealed simple associations between some of those factors and mental health. However, more research is needed to assess interactions among different SDH, SCS, and psychiatric symptoms in more integrated approaches. Besides, new investigations should assess the interplay between SDH and other SCS, social cooperation, social encoding, theory of mind, group belongingness processes, and moral decision making. Although several studies have revealed impairments in those processes in different mental disorders, none has assessed the interactions between them and SDH in predicting psychiatric symptoms. Finally, more research is required to further understand how SCS interventions could reduce the negative impact of certain SDH and to what extent those interventions could reduce the risks of suffering mental problems. To summarize, using a randomized sample selection design and SEM, we found that a complex interplay among social factors, psychiatric antecedents, and somatic conditions seems to be the best combination to predict symptoms of mental illness. In addition, our results highlight the importance of social factors as robust predictors of symptoms of mental illness when they are compared to classical factors. This is especially important given that current studies of predictors of mental symptoms usually underestimate the role of social factors and tend to analyze different factors associated with mental illness in isolated ways^18,19^. Our results may call for the development of new strategies to assess different levels of social dimensions, including individual (SCS) and social contextual (SDH) factors, to predict symptoms of mental illness and thus potentially screen and diagnose psychiatric illness and intervene early.

## Supplementary information

Supplementary material

## Data Availability

The datasets generated and the R scripts for each SEM are available upon request from the corresponding author.
