# Supplementary material for: The role of social cognition skills and social determinants of health in predicting symptoms of mental illness
Source: Transl Psychiatry. 2020 May 26;10:165. doi: 10.1038/s41398-020-0852-4 (PMC7280528; doi:10.1038/s41398-020-0852-4)
Supplement: Supplementary file 1 — Supplementary material [file 41398_2020_852_MOESM1_ESM.docx]

**Supporting Information**

**The role of social cognition skills (SCS) and social determinants of health (SDH) in predicting symptoms of mental illness**

**This file includes:**

1. **Appendix S1. Methods. Data source and study sample**
2. **Appendix S2. Methods. Social Cognition Skills (SCS)**
3. **Appendix S3. Methods. Social Determinants of Health (SDH)**
4. **Appendix S4. Methods. Classical factors. Assessment of psychiatric antecedents**
5. **Appendix S5. Methods. Classical factors. Assessment of cognitive functioning**
6. **Appendix S6. Methods. SEM procedures**
7. **Appendix S7. Supplementary Table 1. General parameters of the latent and observable variables for outcome variables in each SEM.**

**Appendix S1. Methods. Data source and study sample**

The Colombian Mental Health Survey of 2015 (CMHS) is a cross-sectional, descriptive, observational, and population-based study of the (noninstitutionalized) Colombian civil population comprising four age groups: 7-11, 12-17, 18-44 and ≥ 45 years. In this study, we focused on adulthood (population older than 18 and up to 59 years). The overall household response rate was 97.4%, and the individual response rate was 95.1%.

**Structure of the sample design:** The information was collected from a probabilistic sample and based on the master sample of population studies for health of the Ministry of Health and Social Protection in Colombia. The sample was recruited following a stratified methodology in several regions of the country (Atlantic, Oriental, Central, Pacific and Bogotá). The selection of study participants was carried out following a multistage sampling that begins with selecting municipalities, then the blocks of houses in the urban area and rural regions. First, a segment is randomly selected and, within each segment, all the homes. In homes, all households are selected, and from each one, an individual aged ≥ 18 years and a 7-11-year-old child are selected, if there is one in the home.

**Estimation of sample sizes by age group:** The different outcomes of interest were taken into account for each group set: 7-11, 12-18, 18-44, and ≥ 45 years old, considering the prevalence of any disorder according to the Colombian National Mental Health Survey 2003 (CNMHS2). To meet this sample size, 242 segments were selected in each region. Therefore, the representativeness of the study data was established for the age groups assessed in this study (18 and 60 years in each region).

**Appendix S2 Social Cognition Skills (SCS)**

**S2.1 SCS. Emotion Recognition Task**

We assessed facial emotion recognition using the emotion recognition task (EMT)^1^, which comprises of photos of facial expressions featuring six basic emotions (happiness, surprise, sadness, fear, anger, and disgust). We used 12 facial stimuli depicting basic emotions. These images were randomly presented on a 15 inch notebook screen 40-50 cm from the participant. Participants were asked to press a button as soon as they recognized the facial expression and then to identify it from a forced-choice list of six options. The images remained visible until the participant responded. We measured the mean accuracy of overall emotion recognition (maximum one point) and the accuracy for each emotion category. To facilitate the analyses of emotion recognition skills we built two measures following previous studies^2-4^, a positive valence score (happiness and surprise), and a negative valence score (sad, disgust, fear)^2^. This task has been previously validated^5^, is robust to track deficits in patients with neuropsychiatric disorders^6,7,8^ and has been used in other population-based studies^3,4^.

**S2.2 SCS. Empathy for pain task (EPT)**

We used a modified version of a previously reported the Empathy for pain task (EPT)^9^, which evaluates various dimensions of affective empathy in situations involving intentional or accidental harm. The EPT employed here comprises 11 animated scenarios (4 intentional, 4 accidental, 3 neutral) involving two individuals. Each scenario consists of 3 digital color pictures presented in a sequential manner to simulate motion. Durations of the first, second, and third pictures in each animation were 500, 200, and 1,000 ms, respectively. Three types of situations were depicted: (1) intentional harm, in which one person deliberately inflicts pain on another (e.g., one person purposely steps on someone else’s toe); (2) accidental harm, where one person accidentally inflicts pain on another (e.g., one person hits another individual unintentionally by moving his/her chair and not realizing someone was behind him/her); and (3) control or neutral situations involving no harm (e.g., someone hands a flower to another person).

Participants were blind to facial emotional reactions as protagonists’ faces on pictures were not visible. However, bodily expressions and postures provided appropriate information about the victim’s emotional reaction and the agent’s intention.

Participants were asked to respond to five questions for each scenario, i.e., (a) purpose comprehension (was the action done on purpose?), (b) empathic concern (how sad do you feel for the victim?), (c) degree of discomfort (how upset do you feel at what happened?), (d) intention to harm (how serious was the intention?), and (e) punishment (how much punishment does this action deserve?). The question about purpose was answered selecting “Yes” or “No”. The other questions were answered on a visual analogue scale ranging from 0 to 100 –these numbers were not visible to participants. The meaning of extremes of the scale depends on the question. For example, in the question “how sad do you feel for the hurt person?”, one extreme of the bar reads “I feel very sad” and the other extreme reads “I don’t feel sad at all”. We measured accuracy for purpose comprehension questions and ratings for the other questions. Previous behavioral ^9^ and neuroimaging ^10^ studies using similar versions of this task showed that empathy ratings (i.e., empathic concern, discomfort, intention to harm, and punishment) are higher for intentional than for accidental harm, and ratings for both conditions are higher than those for neutral situations. EPT paradigms reliably induce empathic responses^11^, engage putative neural circuits^12^ and triggering automatic sensorimotor resonance between self and others^13^. Importantly, relative to self-report instruments, these tasks can induce more automatic responses^13^. Indeed, self-report measures assess the individuals’ beliefs about their experience of empathy and can be biased by social norms and expectations^14^.

**Appendix S3. Social Determinants of Health (SDH)**

**S3.1 Assessment of social adverse factors**

**S3.1.1 Experiences of discrimination**

Participants were asked if they experienced social isolation and discrimination across their lifespan. They were required to answer the following type of question: Have you ever been discriminated, or isolated from a group due to social/cultural reasons? Participants were further asked to specify whether they had experienced discrimination based on a) age, b) gender, c) sexual orientation, d) level of education, e) type of employment, f) group belonging, g) ethnicity, h) religion, i) socioeconomic status and j) forced displacement.

Item responses were recorded as binary (yes = 1, no = 0). Summing the individual items gave a total score of 10.

**S3.1.2 Violence**

Participants were asked whether they or their close relatives had ever experienced violence, through the following question: Have you ever been a direct or indirect victim of violence/abuse/harassment in your life? Then, they were asked about their experiences of a) physical abuse b) psychological abuse, c) sexual harassment or sexual abuse, d) forced displacement and e) witnessing of violent deaths. Responses were recorded as binary (yes = 1, no = 0). The total number of adverse experiences was used as the variable for this factor. The total of the individual items gave a maximum score of 5.

**S3.1.3 Social stress**

Participants were asked about their difficulties in accessing social well-being resources, through the following question: Have you ever experienced problems, obstacles, or difficulties in accessing social/health/job resources? Then, they stated whether they experienced difficulties in accessing a) educational resources, b) job opportunities, c) health services, d) social participation scenarios, e) political participation and f) recreational activities. Item responses were recorded as binary (yes = 1, no = 0). Summing the individual items gave a total score of 6 points.

**S3.2 Assessment of social protective factors**

**S3.2.1 Social support networks**

Participants were asked about the number of social groups in which they usually participate, through the following type of question: Are you actively engaged in any of the next groups? a) sports groups, b) religion groups, c) academy groups, d) job groups, e) neighborhood groups f) familial groups or g) benefactor groups. Each participant declared their participation in seven social groups by using yes/no questions (yes = 1, no = 0). The total number of groups in which participants were involved was used as the variable for this factor.

**S3.2.2 Assessment of Family Support**

The family support was measured using the 5-item of the Family Adaptation, Partnership, Growth, Affection, Resolve (APGAR) Questionnaire, which collects information on satisfaction with five relevant areas of family function^15^. The Apgar instrument ^15^ has been adapted for the Colombian population^16^. The Family Apgar instrument consists of five questions measuring family members' satisfaction with components considered basic to any family unit and functioning: adaptation, companionship, development, affectivity, and problem-solving ability. Answer options were always (2), sometimes (1) and never (0) - score ranges from 0 to 10; classification varies from 0 to 4, elevated family dysfunction; from 5 to 6, moderate family dysfunction; and from 7 to 10, good family functionality. The score was 0 when participants answered that negatively to the question, and 2 when they reported to have range from 0 to 10, with higher scores indicating greater family support ^15^. The 5 items were as follows: 1) I am satisfied that I can turn to my family for help when something is troubling me; 2) I am satisfied with the way my family discusses things with me and shares problems with me; 3) I am satisfied that my family accepts and supports my wishes to take on new activities or directions; 4) I am satisfied with the way my family expresses affection, and responds to my emotions, such as anger, sorrow, or love; and 5) I am satisfied with the way my family and I share time together. Scores range from 0 to 10, with higher scores indicating greater family support. The Colombian version has been reported as having good reliability, construct validity and a high internal consistency^16,17^. The APGAR has been used as a reliable tool for detecting family support in patients with mental health problems.

**Appendix S4. Assessment of psychiatric antecedents**

Participants were assessed on whether they have ever been diagnosed with a psychiatric disease. In particular, they were assessed for 10 different diseases, including schizophrenia, generalized anxiety disorder, panic disorder, social phobia, specific phobias, anorexia, bulimia, posttraumatic stress disorder, substance use disorder and pathological gambling. Furthermore, the participants also were assessed for the presence of antecedents of four affective diseases including major depression, dysthymia, bipolar disorder, depressive adjustment disorders. To do so, we used the Composite International Diagnostic Interview (CIDI) that follows the criteria of the Diagnostic and Statistical Manual of Mental Disorders (Fifth Edition, Text Revision: DSM-IV-R). The presence of each psychiatric disease was coded using yes/no answers (yes = 1, no = 0). For each disorder participants answered screening questions (a group of two or three questions for each disorder); only when an individual answered affirmatively to those questions did they answer an extra group of questions tracking the presence of a full symptomatology of each disorder. Latent variable of psychiatric antecedents was composed of two observable variables: a) antecedents of general psychiatric disorders (comprised a numerical variable ranging from 0 (without antecedents) to 10 (antecedents of all general psychiatric diseases)); b) antecedents of affective psychiatric disorders (comprised a numerical variable ranging from 0 (without antecedents) to 4 (antecedents of all affective psychiatric diseases).

**Appendix S5. Cognitive functioning**

All participants were evaluated using an executive function battery^18^ measuring (1) motor programming (Luria series, “fist, edge, palm”); (2) conflicting instructions (subjects were asked to hit the table once when the administrator hit it twice, or to hit the table twice when the administrator hit it only once); (3) verbal inhibitory control (modified Hayling test); verbal and design fluency tests were used to assess recall, self-monitoring and cognitive flexibility strategies^19^, and (4) numerical working memory (backward digit span)^19-21^. Previous studies have revealed a negative association between executive functions and symptoms of mental illness^22,23^. These subtasks have been used to successfully detect executive function in clinical^22,23^ and nonclinical populations^24^,^22^. The total score of each subtask ranged from 0 to 3. A 3 score was assigned when participants appropriately answered the items of each subtask.

**Appendix S6. SEM procedures**

**S6.1 Latent and observable variables in each SEM**

Before running each model, we grouped the observable (measured) variables (all observable measures are described at the Instrument section) to build latent (unmeasured) variables, which fitted to the observable variables following theoretical principles^25,26^. To this end, we first tested whether each observable variable of a theoretical construct, actually reached significant regressor scores in predicting the latent variable. Following previous procedures^25,26^ we maintained only those theoretical latent variables in which each observable variable reached significant values. Thus, we built a latent variable named Mental Symptoms based on the sub-scores of depression, anxiety and other symptoms including sensory-perceptual symptoms in the SRQ.

Moreover, we created a latent variable for the SDH (including a latent variable named Social Adverse Factors and observable variables named Familial Support and Social Support Networks). Similarly, we created a latent variable for psychiatric factors (Psychiatric antecedents), a latent variable for physical-somatic factors (Physical somatic problems), and a latent variable for cognitive factors (Cognitive functioning). For further information, see Table 1.

**S6.2 Parameters of SEM**

Following procedures reported in previous studies, we used the three criteria to measure the goodness-of-fit of each model including the X2, the comparative fit index (CFI) and the root mean square error of approximation (RMSEA) methods ^25,26^. Regarding to the χ2, a smaller score indicates that the theoretical model is more suitable for the actual data, and the non-significant (*p* > 0.05) χ2 indicates that the theoretical model is well fitted to the sample data. With respect to the CFI, it is expected that the model reached values above 0.85 (this index ranges from 0 to 1), which is considered an indicator of good modeling fitness ^25,26^. Finally, values below 0.05 in the RMSEA suggest that the model is close to fit ^25,26^. The YB χ2 statistic is used as a fit index (the higher the values from zero, the larger the significance; i.e., *p*-value should be > 0.05). The Robust CFI is an index (0 to 1) that assesses the extent to which the specified model improves fit over the null model (values > 0.90 considered as acceptable fit, values in the range of 0.95-0.99 considered as excellent fit, and a value of 1 considered as exact fit) ^27,28^. The Robust RMSEA indicates the discrepancies between the sample variance-covariance matrix and the model implied variance-covariance matrix (values > 0.08 considered as poor fit, values in the range of 0.05-0.08 considered as adequate fit, and values ≤ 0.05 considered as good fit)^29,30^. In all models we tested three increasingly restrictive hypotheses about factorial invariance across sex: configural invariance, weak invariance, and strong invariance. The configural invariance hypothesis assumes that both the number of factors and the correspondence between factors and the measured variables are the same across groups. In this model, all parameters are freely estimated in each group (except for those used to identify the factor structure in each group). If the configural invariance model fits the data well, the next step consist in examining a model with factor loadings being invariant across groups (i.e., weak invariance). The strong invariance model assumes weak invariance and requires equal unstandardized intercepts (i.e., the mean of measured variables) over the groups^31^. Support for the weak invariance hypothesis and the strong invariance hypothesis requires small changes in the Robust CFI index (i.e., a value of ΔCFI smaller than or equal to 0.01 in a successive comparison: configural invariance vs. weak invariance, and weak invariance vs. strong invariance)^31^. Retaining the weak invariance hypothesis enables formal comparisons of the variance-covariance of factors across groups, and a strong factorial invariance is a precondition for comparing latent factor means across groups ^29,31^.

**S6.3. Comparisons among tested SEM**

Akaike and a sample-size adjusted Bayesian (BIC) were used to analyze the goodness-of-fit indices of each model (see Supporting Information S5.3). The AIC and BIC have been shown to be a useful procedure in selecting the best model from a group of competing models, in particular when complex models are tested^32^. These techniques have been previously used to compare models of different social cognition and social predictors^33^.

**S6.4 Sex analyses in all SEMs**

In models assuming strong factorial invariance, we set the factor means for the male group to zero, thus specifying males as the reference group. Therefore, the model estimated differences of females with respect to the reference group. Figures only show the intercepts of those latent variables in which the estimated differences of females were significantly different from males.

**Models comparison**

We tested various global-integrated SEMs which included all factors but in this case we fixed to 0 a group of factors in each model and left free the rest of factors to calculate its impact in predicting mental symptoms. Thus we ran: a) a SEM fixing to 0 the SCS factor (to measure predictive scores of mental symptoms of rest of factors including SDH, psychiatric, physical-somatic and executive functioning factors) b) a SEM fixing to 0 SDH factor (assessing the predictive scores of the rest of factors); c) a SEM fixing to 0 the psychiatric, physical-somatic and executive functioning factors (assessing the predictive scores of the rest of factors); and d) a SEM fixing to 0 SCS and SDH (assessing the predictive scores of the rest of factors). Akaike and sample-size adjusted Bayesian (aBIC) criteria were used to compare the goodness-of-fit indices between models.

The AIC and BIC allow the comparison between models of different complexity and are derived from the log likelihood. The model with lowest AIC and BIC is preferred. We checked if the elimination of a fixed effect or a random effect left a better fit with respect to AIC, BIC and log-likelihood criteria.

Data processing and all analyses were made using R (R Development Core Team, 2010). The specific R packages used were the nlme package (the R Development Core Team, 2010), the lattice package, the ggplot2 package, and the reshape package.

**Appendix S7.** In all models, the factor loadings of all observable and latent variables were statistically significant at *P*<0.001. In all models the estimated intercepts of the latent variables of emotion recognition, empathy for pain and executive functioning did not reveal sex differences, however, the intercepts of latent variables of mental symptoms, psychiatric antecedents and physical somatic problems indicated that females showed higher scores in those latent variables than males (differences in the aforementioned intercepts are represented as triangles in Figures 1-5).

**Appendix S7. Supplementary Table 1. General parameters of the latent and observable variables for outcome variables in global-integrated-SEM.**

| **Factors** | **Latent Variables** | **Observable**  **Variables** | **Estimates**  **F:M** | **Standard Error**  **F:M** | **Z-value**  **F:M** | **P(>\|z\|)**  **F:M** | **Standardized Scores**  **F:M** | | |  |  |  |  |
| --- | --- | --- | --- | --- | --- | --- | --- | --- | --- | --- | --- | --- | --- |
| **Outcome variables** | **Mental Symptoms** | **Depression**  **Anxiety**  **Other Symptoms** | 1.28: 1.28  1.11: 1.11  0.36: 0.36 | 0.07: 0.07  0.05: 0.05  0.02: 0.02 | 18.2: 18.2  20.4: 20.4  15.7: 15.7 | 0.0: 0.0  0.0: 0.0  0.0: 0.0 | 0.87: 0.89  0.78: 0.80  0.50: 0.45 | | |  |  |  |  |
| **Social cognition skills (SCS)** | **Emotion Recognition** | **Positive emotions**  **Neutral-negative**  **emotions** | 0.58: 0.58  0.39: 0.39 | 0.04: 0.04  0.03: 0.03 | 12.2: 12.2  12.3: 12.3 | 0.0: 0.0  0.0: 0.0 | 0.62: 0.61  0.61: 0.62 |  |  | |  |  |  |
|  | **Empathy for Pain** | **Intentional**  **Affective**  **Accidental**  **Affective** | 0.69: 0.69  1.23: 1.23 | 0.04:0.04  0.06: 0.06 | 17.3: 17.3  20.6: 20.6 | 0.0: 0.0  0.0: 0.0 | 0.46: 0.44  0.96: 0.93 | | |  | |  |  |
|  |  | **Intentional**  **Cognitive**  **Accidental**  **Cognitive** | 0.27: 0.27  0.66: 0.66 | 0.03: 0.03  0.04: 0.04 | 8.6: 8.6  15.7:15.7 | 0.0: 0.0  0.0: 0.0 | 0.21: 0.21  0.45: 0.45 | | |  | |  |  |
| **Social Determinants of Health (SDH)** | **Social Adverse**  **factors** | **Discrimination experiences Violence**  **experiences**  **Social Stress (restricted access to social resources)** | 0.76: 0.76  0.10: 0.10  0.53: 0.53 | 0.06: 0.06  0.01: 0.02  0.05: 0.05 | 11.4: 11.4  5.2: 5.2  9.4: 9.4 | 0.0: 0.0  0.0: 0.0  0.0: 0.0 | 0.59: 0.56  0.19: 0.17  0.36: 0.33 | | |  | |  |  |
| **Psycho-physical**  **Factors** | **Psychiatric**  **Antecedents** | **General Antecedents**  **Affective**  **Antecedents** | 0.48: 0.48  0.48: 0.48 | 0.03: 0.03  0.03: 0.03 | 14.03  14.03 | 0.0: 0.0  0.0: 0.0 | 0.95: 1.00  0.84: 0.78 | | |  | |  |  |
|  | **Physical Somatic**  **Conditions**  **Cognitive Functioning** | **Chronic**  **Diseases**  **Motor planning**  **Conflictive Instructions** | 0.23: 0.23  0.40: 0.40  0.34: 0.34 | 0.01: 0.01  0.02: 0.02  0.01: 0.01 | 35.89  19.7: 19.1  21.5: 21.5 | 0.0: 0.0  0.0: 0.0  0.0: 0.0 | 1.00: 1.00  0.63: 0.59  0.65: 0.62 | | |  | |  |  |
|  |  | **Inhibitory verbal control**  **Backward digit spam** | 0.67: 0.67  0.34: 0.34 | 0.04: 0.04  0.02: 0.02 | 15.5: 15.5  16.4: 16.4 | 0.0: 0.0  0.0: 0.0 | 0.42: 0.36  0.44: 0.40 | | |  | |  |  |

**References**

1 Ekman, P. Facial expressions. *Handbook of cognition and emotion* **16**, e320 (1999).

2 Stel, M. & Van Knippenberg, A. The role of facial mimicry in the recognition of affect. *Psychological Science* **19**, 984 (2008).

3 Molinero Caparrós, C., Bonete, S., Gómez-Pérez, M. & Calero, M. *A normative study of the Ekman 60-faces test in Spanish adolescents*. Vol. 23 (2015).

4 Biehl, M. *et al.* Matsumoto and Ekman's Japanese and Caucasian Facial Expressions of Emotion (JACFEE): Reliability data and cross-national differences. *Journal of Nonverbal behavior* **21**, 3-21 (1997).

5 Young, A. W. *et al.* Facial expression megamix: Tests of dimensional and category accounts of emotion recognition. *Cognition* **63**, 271-313 (1997).

6 Ibanez, A., Kuljis, R. O., Matallana, D. & Manes, F. Bridging psychiatry and neurology through social neuroscience. *World Psychiatry* **13**, 148-149 (2014).

7 Cotter, J. *et al.* Social cognitive dysfunction as a clinical marker: A systematic review of meta-analyses across 30 clinical conditions. *Neurosci Biobehav Rev* **84**, 92-99, doi:10.1016/j.neubiorev.2017.11.014 (2018).

8 Gonzalez-Gadea, M. L. *et al.* Emotion recognition and cognitive empathy deficits in adolescent offenders revealed by context-sensitive tasks. *Frontiers in human neuroscience* **8**, 850 (2014).

9 Baez, S. *et al.* Integrating intention and context: assessing social cognition in adults with Asperger syndrome. *Frontiers in human neuroscience* **6**, 302, doi:10.3389/fnhum.2012.00302 (2012).

10 Baez, S. *et al.* Orbitofrontal and limbic signatures of empathic concern and intentional harm in the behavioral variant frontotemporal dementia. *Cortex; a journal devoted to the study of the nervous system and behavior* **75**, 20-32, doi:10.1016/j.cortex.2015.11.007 (2016).

11 Decety, J. & Cacioppo, S. The speed of morality: a high-density electrical neuroimaging study. *Journal of neurophysiology* **108**, 3068-3072, doi:10.1152/jn.00473.2012 (2012).

12 Akitsuki, Y. & Decety, J. Social context and perceived agency affects empathy for pain: an event-related fMRI investigation. *NeuroImage* **47**, 722-734, doi:10.1016/j.neuroimage.2009.04.091 (2009).

13 Jackson, P. L., Rainville, P. & Decety, J. To what extent do we share the pain of others? Insight from the neural bases of pain empathy. *Pain* **125**, 5-9, doi:10.1016/j.pain.2006.09.013 (2006).

14 Decety, J. The neuroevolution of empathy. *Annals of the New York Academy of Sciences* **1231**, 35-45, doi:10.1111/j.1749-6632.2011.06027.x (2011).

15 Smilkstein, G. The family APGAR: a proposal for a family function test and its use by physicians. *J fam pract* **6**, 1231-1239 (1978).

16 Ariza, L. M. F., Durán, M. C. A., Cubillos, Z. J. D. & Arias, A. *Consistencia interna y análisis de factores de la escala APGAR para evaluar el funcionamiento familiar en estudiantes de básica secundaria*. (Red Revista Colombiana de Psiquiatría, 2006).

17 Nan, H., Lee, P. H., Ni, M. Y., Chan, B. H. & Lam, T.-H. Effects of depressive symptoms and family satisfaction on health related quality of life: the Hong Kong FAMILY study. *PLoS one* **8**, e58436 (2013).

18 Nunes, D. *et al.* INECO frontal screening: a tool to assess executive functions in depression. *Psicol. clin.* **26**, 177-196 (2014).

19 Delis, D. C., Kramer, J. H., Kaplan, E. & Holdnack, J. Reliability and validity of the Delis-Kaplan Executive Function System: an update. *Journal of the International Neuropsychological Society* **10**, 301-303 (2004).

20 Burgess, P. W. & Shallice, T. Response suppression, initiation and strategy use following frontal lobe lesions. *Neuropsychologia* **34**, 263-272 (1996).

21 Gilbert, S. J. & Burgess, P. W. Executive function. *Current Biology* **18**, R110-R114 (2008).

22 Baez, S. *et al.* Primary empathy deficits in frontotemporal dementia. *Front Aging Neurosci* **6**, 262, doi:10.3389/fnagi.2014.00262 (2014).

23 Baez, S., García, A. M. & Ibanez, A. in *Social Behavior from Rodents to Humans* 379-396 (Springer, 2016).

24 Tobón, C. *et al.* Psychiatric, cognitive and emotional profile in ex-combatants of illegal armed groups in Colombia. *Revista colombiana de psiquiatria* **45**, 28-36 (2016).

25 Hoe, S. L. Issues and procedures in adopting structural equation modeling technique. *Journal of applied quantitative methods* **3**, 76-83 (2008).

26 Schreiber, J. B., Nora, A., Stage, F. K., Barlow, E. A. & King, J. Reporting structural equation modeling and confirmatory factor analysis results: A review. *The Journal of educational research* **99**, 323-338 (2006).

27 Xia, Y. & Yang, Y. RMSEA, CFI, and TLI in structural equation modeling with ordered categorical data: The story they tell depends on the estimation methods. *Behav Res Methods* **51**, 409-428, doi:10.3758/s13428-018-1055-2 (2019).

28 Ullman, J. B. Structural equation modeling: reviewing the basics and moving forward. *J Pers Assess* **87**, 35-50, doi:10.1207/s15327752jpa8701_03 (2006).

29 Savalei, V. On the Computation of the RMSEA and CFI from the Mean-And-Variance Corrected Test Statistic with Nonnormal Data in SEM. *Multivariate Behav Res* **53**, 419-429, doi:10.1080/00273171.2018.1455142 (2018).

30 Savalei, V. & Kolenikov, S. Constrained versus unconstrained estimation in structural equation modeling. *Psychol Methods* **13**, 150-170, doi:10.1037/1082-989X.13.2.150 (2008).

31 Putnick, D. L. & Bornstein, M. H. Measurement Invariance Conventions and Reporting: The State of the Art and Future Directions for Psychological Research. *Dev Rev* **41**, 71-90, doi:10.1016/j.dr.2016.06.004 (2016).

32 Vrieze, S. I. Model selection and psychological theory: a discussion of the differences between the Akaike information criterion (AIC) and the Bayesian information criterion (BIC). *Psychological methods* **17**, 228 (2012).

33 A, I. *et al.* Motor-language coupling: direct evidence from early Parkinson's disease and intracranial cortical recordings., doi:10.1016/j.cortex.2012.02.014 (2013).
